# Evaluation of complex multi-physics phenomena at gas diffusion electrodes during high-pressure water electrolysis with AEMs

**DOI:** 10.1038/s41598-025-05216-5

**Published:** 2025-07-10

**Authors:** Erik Delp, Rakesh Mishra, Enno Wagner

**Affiliations:** 1 Frankfurt University of Applied Sciences, Frankfurt, Germany; 2 School of Computing and Engineering, University of Huddersfield, Huddersfield, United Kingdom

**Keywords:** Alkaline membranes, AEM, Gas diffusion electrodes, High pressure electrolyzer, Impedance analysis, OER, HER, Wartburg element, Energy, Materials science, Energy science and technology, Energy harvesting, Energy infrastructure, Energy storage, Fuel cells, Renewable energy, Thermoelectric devices and materials, Chemical engineering, Electrical and electronic engineering, Engineering, Energy infrastructure, Power distribution

## Abstract

The alkaline water electrolysis is a well-established process for producing green hydrogen from renewable energy sources. With up-to-dateAEM electrolyzers, electrochemical gas compression can be realized with water electrolysis and ion pumping membranes, to avoid costly mechanical compression. In this experimental study, we researched an electrolyzer cell with a strong metal structure, for internal pressure difference of up to 100 bar. Micro-porous gas diffusion electrodes containing non-precious nickel catalysts as well as different separators, alkaline membranes and AEMs have been investigated in the range of 300 to 800 mA cm^− 2^. For one preferred AEM, characteristics are shown for hydrogen pressures between 20 and 80 bars, while the anode remains at ambient 1 bar. Impedance spectroscopy diagrams are used to display the individual cell components: the ohmic resistance of the AEM and the complex impedances of both electrodes. Therewith, we could visualize the complex multi-physics phenomena and show that the oxygen electrode works as a Wartburg-element, especially due to higher diffusion rates and therewith entropy production during bubble formation.

## Introduction

By applying a voltage to two electrodes immersed in an aqueous solution, water can be split electrochemically, as was discovered at the beginning of the 19th century. Especially alkaline electrolyzer came up early, where the hydrogen evolution reaction (HER) takes place at the negative cathode

$$\:2\:{H}_{2}O\:+2\:{e}^{-}\to\:2\:O{H}^{-}+{H}_{2}$$

while the oxygen evolution reaction (OER) happens at the positive anode.

$$\:2\:O{H}^{-}\to\:\:{H}_{2}O\:+2\:{e}^{-}\:+\:\frac{1}{2}\:{O}_{2}$$

Hereby, simple Nickel mesh electrodes were separated by asbestos diaphragms which allow no pressure difference between the two gas sides.

Some 200 years later, water electrolysis is a promising method of using chemical energy to store excess capacity of intermittent electrical energy from renewable sources^1–5^. For example, hydrogen produced by electrolysis can be used in combustion processes^6^, methanation of CO_2_^5,7,8^ and fuel cell regeneration^9,10^. Fuel cells are an exciting new technology for vehicle drive applications that could provide zero-emission driving using green hydrogen^11^. Our future energy systems will most definitely include green hydrogen produced from water electrolysis using renewable electricity. Generally, it is recommended that electricity from wind and solar power should be used - whenever it is possible - in the most effective manner, like in electric drives, battery cars or heat pumps. However not all the operations are amenable to easy electrification. In the chemical industry there are various processes which requires gaseous energy carriers with high power density like natural gas or hydrogen. Along with substituting fossil fuels, hydrogen as a fuel is in use in the processes like the production of ammonia and fertilizer which uses up to 50% of the currently global hydrogen production^12^. The ammonia und fertilizer production needs hydrogen with a pressure range between 100 and 200 bar, next to it the steal production, which will have a high need of hydrogen needs 5–10 bar and the gas grid to transport the power is at 70 bar and gas caverns to store the gas long-term are using a pressure range of 130 to 200 bar^13^. With those, the biggest needs accept of the transport sector could be addressed with a pressure range of 10 to 200 bar.

For long distances vehicles, fuel cell drives with hydrogen are a promising alternative to batteries. Especially for storage capacities above 60 kWh, fuel cell drives have a lower impact on the environment than batteries, when the entire life cycle (including production, use and disposal) is considered^14^ But, for long distance vehicles, high volumetric energy densities are required, and therewith hydrogen pressures of around 700 bar^15,16^. Due to the thermodynamics of isothermal mechanical compression, a temperature increase affects the amount of energy required to compress the gas. In contrast to that, low temperature water electrolysis are advantageous^17,18^. Electrochemical hydrogen pre-compression can prevent further mechanical compression and the associated maintenance problems and investment costs by eliminating or reduce the need of a compressor. The demand of precompressed hydrogen is quite high.

For electrochemical compression, the hydrogen gas evolution by water electrolysis can be conducted under pressure^19–22^. Generally, the methods of water electrolysis involves the use of liquid or solid electrolytes, whose conductivity is provided by protons or hydroxide ions^11^. The solid acids known as polymer electrolyte membranes (PEMs)^23–28^ are essentially positive charged protons that are movable in the aqueous phase, while the negative charged acid residue anion is fixed to the polymer structure of the membrane. The power density of water electrolysis using solid polymer electrolytes is superior to that of conventional liquid alkaline electrolytes across a wider dynamic load range^1,29–32^. Oxygen pressurization is no longer necessary since solid polymer electrolytes are mechanically robust and can withstand higher cathodic than anodic pressure^5,17,18,20^. Commercial PEM electrolyzes are working with a hydrogen outlet pressure of typically 30–40 bar while the oxygen is released at ambient pressure^33^. Some special electrolysis prototypes can produce even 200 bars and more^34,35^. However, precious metals like platinum and iridium must be used as catalysts in PEM electrolyzers, because they are acidic, where nearly all metal catalysts are unstable due to a high oxidative potential^36^. Moreover, the global supply of platinum and iridium is severely constrained^37–39^. Consequently, the production of green hydrogen is inadequate to meet global demand^40^. The implementation of alternative technologies, such as AEM electrolysis, is imperative. In addition to the availability of materials, electrolysis offers economic advantages by avoiding the use of acidic environments.

**Fig. 1:**
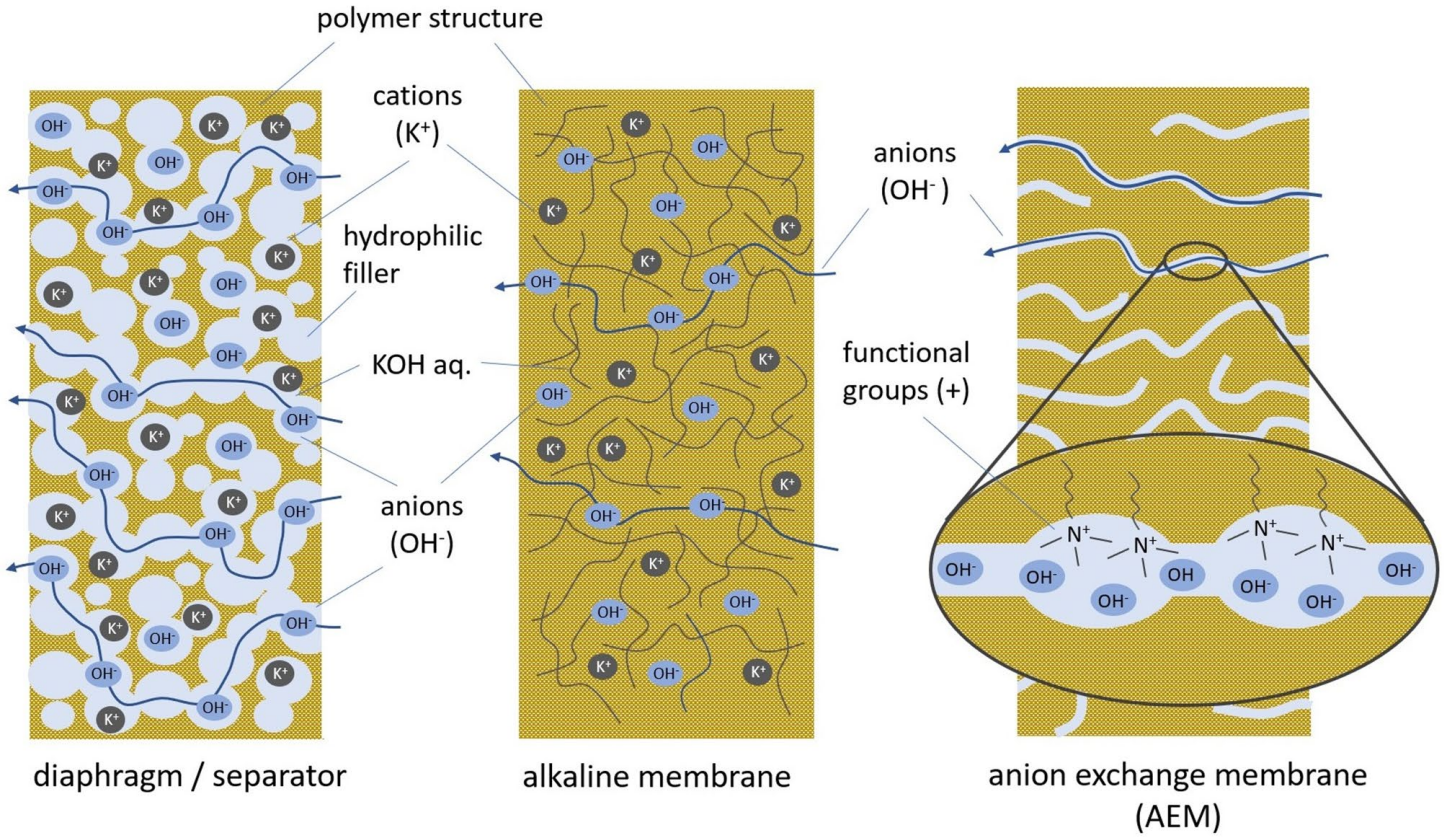
Schematic illustration of separators, alkaline membranes and anion exchange membranes (AEM) according to^41^.

In alkaline environment, a variety of nonprecious metals such as nickel, carbon, iron and cobalt can be used as catalysts, while the electrochemical efficiency is generally higher than in acidic cells. These advantages have led to an impressive development in recent decades, as shown in Fig. 1. In conventional alkaline electrolyzers, simple separators are used which have a stable support structure (asbestos or high performance plastics), with some hydrophilic filling material (e.g. titanium oxide TiO_2_). They are highly conductible for hydroxide ions when soaked with concentrated KOH, but they cannot hold any pressure difference. Alkaline membranes have a closed structure like a foil and can withstand high differences of gas pressure. On the other hand, the resistance for ion flows is relatively high and the carbonate formation with free potassium ions (K^+^) can be hindering. Modern anion exchange membranes (AEMs) combine two advantages: they are highly conductive for hydroxide ions and they are tight enough to build a remarkable pressure difference^41^.

The chemical structure of AEMs is comparable to PEMs, but with opposite charge conditions. Here, the positive functional group (e.g. quaternary ammonium group NH_3_^+^) is fixed to the polymer backbone of the membrane, while the hydroxide ions (OH^−^) are mobile. But, the long-term stability of these chemical compounds of all anion exchange membranes (AEMs) needs improvement and is still a challenge^42–46^. In^47^ for example, the degradation of quaternary ammonium groups due to Hofmann elimination in concentrated KOH is described. Especially, functional groups based on imidazole, which are connected to aromatic polymer backbones (e.g. the AEM Sustainion^®^ from Dioxide Materials) seem to be stable for a longer duration at temperatures above 60 °C^48^.

Another crucial point of water electrolysis is the inefficient evolution of gas bubbles in aqueous electrolyte solution or pure water. We have shown before that the formation of gas bubbles at the surface of simple nickel mesh has a high significance for the overvoltage at the oxygen electrode, in contrast to efficient gas diffusion electrodes with no bubble formation^49^.

Using a reversible alkaline fuel cell in the classic design with an electrolyte gap at ambient pressure, detailed measurements with highly-efficient gas diffusion electrodes, different catalysts (Raney-nickel, graphite, platinum and Raney-silver) were conducted. Herewith, very high electrochemical efficiencies up to 100% for the hydrogen production could be obtained^50^.

But, for the development of electrochemical compressors with ion-pumping membranes, a robust electrolyzer cell in zero-gap design must be realized. It is important to find stable alkaline membranes or AEMs that that can withstand internal pressure difference up to 100 bars. Under this extreme pressure conditions, especially the diffusive mass transport within the membrane and the gas diffusion electrodes should be analyzed in detail using impedance spectroscopy.

## Materials and methods

In this experimental work, we studied alkaline membranes and AEMs which are sandwiched between porous gas diffusion electrodes (GDE) using a high contact pressure. The design concept of the electrochemical cell allows the evolution of hydrogen by diffusion into dry gas spaces, while the oxygen evolution takes place in flooded flow structures with aqueous electrolyte.

**Fig. 2:**
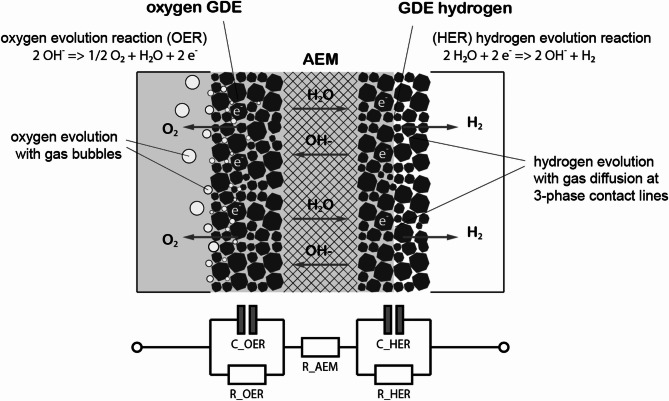
Schematic representation of an anion exchange membrane (AEM) sandwiched between gas diffusion electrodes (GDE). The impedance model is shown in the lower part of the figure, representing the equivalent network with impedances for the AEM and the two electrodes.

As shown in Fig. 2, there are three main regions which must be considered: the AEM in the center is highly conductive for hydroxide ions (OH^−^) and allows the diffusion of water molecules. Furthermore, the membrane is gas-tight and can withstand a high differential pressure between the oxygen and the hydrogen side. Modelling of the membrane is done using a simple electrical resistance R_AEM_ for the ion conductivity that is essentially temperature dependent. The behavior of the gas diffusion electrodes (GDE) is fundamentally different. Here, the complex physical phenomena of the phase transition from the liquid to the gaseous state takes places, while exergy is dissipated, and entropy is being produced. Here, all these complex mechanisms are summarized for the respective electrode using linear electrical resistances (R_OER_ / R_HER_), which are connected in parallel with the respective capacitors (C_OER_ / C_HER_). The combination of a resistor with a capacitor leads to an impedance in the complex level, represented by a pointer with amplitude and phase. Since the impedance is strongly dependent on the excitation frequency, it can be viewed as a PT1 element, typically represented by a semicircle in the Nyquist diagram.

For the experimental evaluation of gas diffusion electrodes and their underlying physical mechanisms, the impedance of the OER and HER are carefully characterized. To explain the complex multi-physics behaviour of the porous nickel structure, a constant phase element is required for the electrode. The constant phase element (CPE) is an important electrochemical modelling element used in the characterization of electrode systems^51–53^. In contrast to an ideal capacitor, the CPE represents a non-ideal, frequency-dependent capacitance and enables a more precise description of diffusion processes and non-linearity`s on electrode surfaces.

Hereby, the research question must be answered, if the higher inefficient caused by bubble formation will be measurable by an impedance with stronger parts of a Wartburg element. The influence of increasing hydrogen gas pressure on the OER and HER should be also analyzed and discussed.

### Safety considerations

It is evident that the safety requirements escalate in proportion to the gas pressure, particularly in the presence of hydrogen and oxygen. Hydrogen is susceptible to combustion, especially when in close proximity to oxygen. Similarly, pressurized oxygen can ignite combustion, particularly in the presence of impurities. In addition, certain components of the electrolyzer cell are typically manufactured from titanium, which, under conditions of high titanium purity, a rough surface, and oxygen pressures reaching a few bar (a function of the (ignition) temperature, among other parameters), may result in self-ignition^54^. However, there have been no documented incidents of self-ignition during electrolyzer operation. This may be attributed to the presence of liquid water and/or the protective oxide layers on the titanium and stainless steel (V4) surfaces. For both differential and balanced pressure operation, gas crossover through the membrane^55^ poses a safety concern at high gas pressures, as per Fick’s law. In particular, the hydrogen contamination of the oxygen gas must be considered a critical factor, and a combustible atmosphere must be avoided^56^. The lower explosion limit (LEL) of hydrogen in oxygen is at about 4 mol% under standard conditions, it increases with pressure and slightly decreases with temperature^57^. In order to minimize the potential hazards associated with the test rig, a recombination catalyst was incorporated into the oxygen path. Concurrently, a hazard and operability (HAZOP) risk assessment was conducted, and the findings were integrated into the test stand control system.

## Alkaline pressure electrolyzer design

For realizing the above introduced concept, an electrolyzer cell was designed with strong metal plates to withstand high internal pressure of up to 100 bar. It has a special sealing to allow a high differential pressure over alkaline membranes and anion exchange membranes (AEM) in combination with gas diffusion electrodes (GDE).

**Fig. 3:**
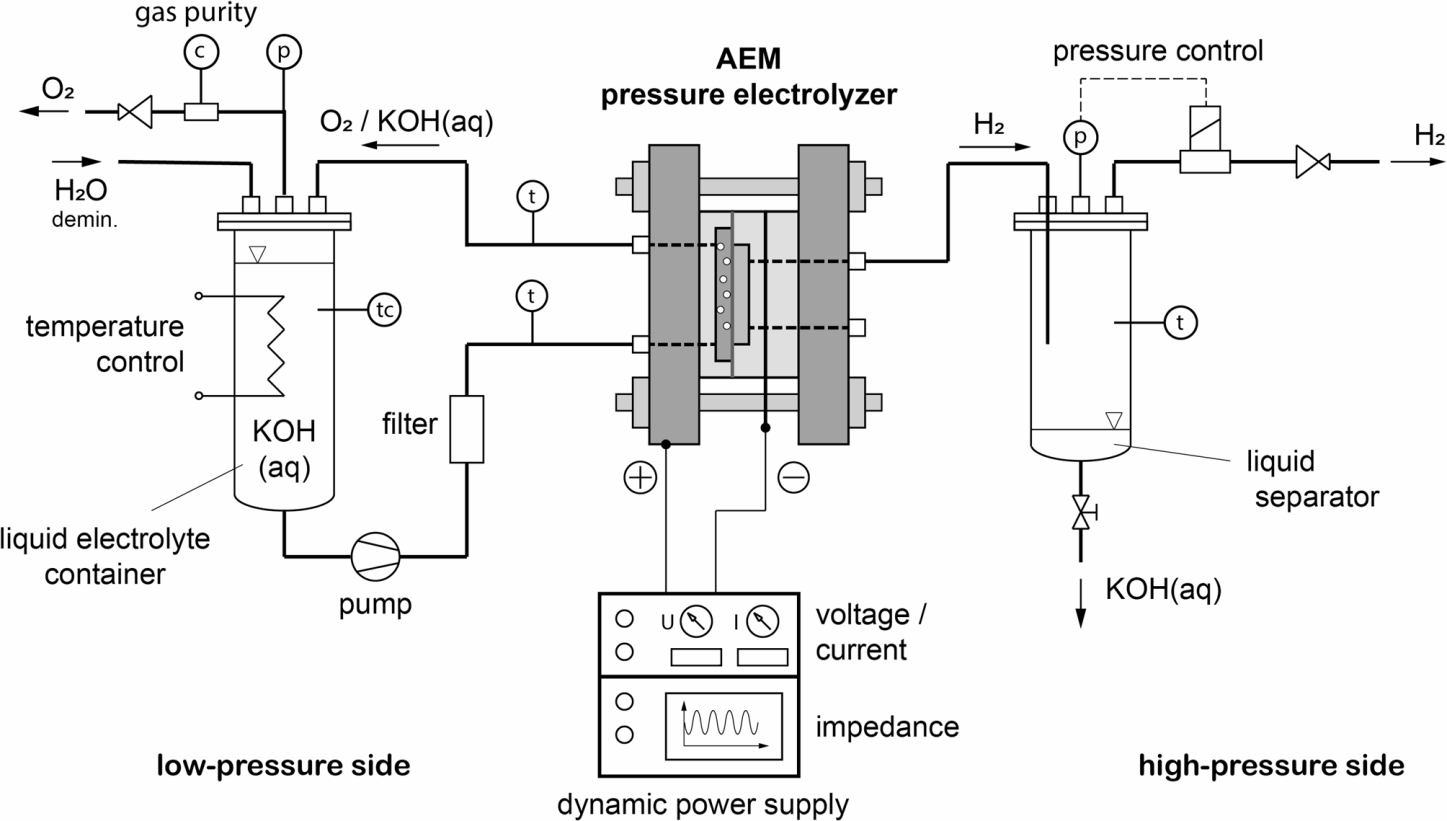
process flow sheet of the AEM pressure electrolyzer in a test stand at the Frankfurt University of Applied Sciences.

The Frankfurt University of Applied Sciences improved an early design from the ACTA Company to create the zero-gap design for anion exchange membrane electrolyzer. It was set up to test various electrodes and membranes in the Lab of Fuel Cell Technology at Frankfurt University of Applied Sciences. To achieve pure hydrogen and provide the opportunity to manufacture pre-compressed hydrogen gas, the design is similar to a PEM electrolyze. Figure 3 provides a schematic illustration to demonstrate the zero-gap operating concept (please do not scale from this picture). A separator or alkaline membrane is the one that guarantees the movement of water and hydroxide ions (OH-). Gas diffusion electrodes are squeezed on each side of this membrane with the assistance of heavy steel endplates and metallic support structures. The support structures were made from welded-together stainless-steel meshwork of various sizes. To provide a solid electric connection, the side that faces the electrode is polished. A synthetic material framework constructed of polypropylene (PP) encloses the support structures, which include the electrodes and membrane, and is sealed with several gaskets. Comparing this AEM cell design to other popular ones, the ohmic resistance of the cell is significantly reduced, but highly depending on the tolerances to achieve a proper electrical and chemical transport.

Figure 2 shows that the hydrogen side of the electrolyzer is nearly dry whereas the oxygen side is completely submerged in liquid electrolyte. In terms of mass transfer in the electrochemical process, the generated gas at the HER can permeate freely into the dry support structure, but is constrained by the amount of Liquid passing through the membrane. Gas bubbles need to develop also at the oxygen side, which is a complex and energy-intensive process. On the other hand, water needs to diffuse across the membrane against the current of the hydroxide ions because water splitting occurs at the HER. The negative impact of mass transportation could reduce the benefits of a thin membrane and the resulting decrease in electrolyte resistance.

## Experimental setup

The DC power source for the electrolyzer in this setup is a four-quadrant NL series power supply from Höcherl & Hackl GmbH (model NL1V10C20 Standard), which provides a constant current in the range − 20 A to 20 A or a constant voltage from − 1 V to 10 V. The accuracy of the H&H power supply is ± 0.2% of the set value and ± 0.05% of the total range. This means a maximum of ± 0.0048 V and ± 0.005 V. The maximum deviation is therefore ± 0.0098 V. The losses via cables and terminals are compensated by additionally attached sense cables and can therefore be neglected. The impedance of the cells was measured using an electrochemical workstation from the German company ZAHNER (model Zennium Pro). It allows a frequency range from 10 µHz to 8 MHz, with a current from − 3to + 3 A, in a voltage range from − 15 to + 15 V. Connected directly to the Höcherl & Hackl four-quadrant supply, the power values are raised to the same level as the four-quadrant supply, without sacrificing precision. The pressure on the hydrogen side is controlled and measured by a pressure maintenance valve from Bronkhost. The pump used is a KNF diaphragm liquid pump, model FP 1.150, for the KOH solution with a pumping capacity of up to 1.1 l/min. The temperature of the KOH is controlled by a Lauda Bar thermostat (model eco silver) and measured by a PT100 sensor, which is added to the KOH stream immediately before and after the electrolyser. Heating mats with Omron temperature controllers (ESCC-TQX3A5M-003) are also installed at the end plates. Table 1 lists the testing circumstances. The KOH concentration was subject to daily monitoring through the implementation of a titration procedure, with adjustments being made as required to ensure its accuracy. The target concentration was consistently entered with a margin of error of ± 5% For pressure control and pressure measurement the A P-512 C-M 10 A-ABD-33-V digital regulator from Bronkhost was used. This allows a control range of 2 to 100 bar and a flow rate of around 10 l h^− 1^ hydrogen. This measurement has an accuracy of 0.5% of the final value. The measurement therefore has a maximum deviation of 0.5 bar at 100 bar pressure. The hydrogen concentration was measured with an TCD3000 sensor from Archigas, which was applied next to the pressure sensor of the O_2_ loop. It can measure H_2_-quantity’s from 10ppm exact.

**Table 1:** Testing parameters of the different separators, membranes and AEMs.

company Membrane	Sustainion X37	FuMATech FAS50 / 30		AGFA UTP 220		Celgard 5550		FuMATech FAAM 20		Tokuyama A201	
Type	AEM	AEM		Separator		Separator		Membrane		AEM	
KOH	1 mol l-1	1 mol l-1		6 mol l-1		6 mol l-1		6 mol l-1		1 mol l-1	
Temperature used	70 °C	70 °C		70 °C		70 °C		70 °C		70 °C	
Temperature recommended	60 °C	40 °C		130 °C		70 °C		60 °C		50 °C	
Differential Pressure	Up to 10 bar	Up to 100 bar		Up to 10 bar		Up to 4 bar		Up to 60 bar		Up to 60 bar	
Pump speed	0,5 l/min	0,5 l/min		0,5 l/min		0,5 l/min		0,5 l/min		0,5 l/min	
Pretreatment of the Membrane	24 h soaking in 1 mol l-1 KOH by room temp	24 h soaking in 1 mol l-1 by KOH room temp		Not needed		Not needed		24 h soaking in 6 mol l-1 KOH by room temp		24 h soaking in 1 mol l-1 KOH by room temp	

The devices used here, i.e. the four-quadrant power supply unit, the temperature control and also the lye flow and lye strength, result in the following deviations in the control parameters. These are shown in Table 2.

**Table 2:** Measurement accuracy of the testing rig.

	deviation		Percentage deviation
Molarity	$\:\pm\:0.1$mol l^− 1^	$\:\pm\:10\%$ AEM $\:\pm\:1.6\%$ Separator / Membrane	
Voltage	± 0.0098 V	$\:\pm\:0.2\%$	
Temperature recommended	$\:\pm\:3^\circ\:C$	$\:\pm\:4.2\%$	
Differential Pressure	$\:\pm\:1\:bar$	$\:\pm\:1\%$	
Pump speed	$\:\pm\:0.01\:l{\:m}^{-1}$	$\:\pm\:2\%$	

## Gas diffusion electrodes

**Fig. 4:**
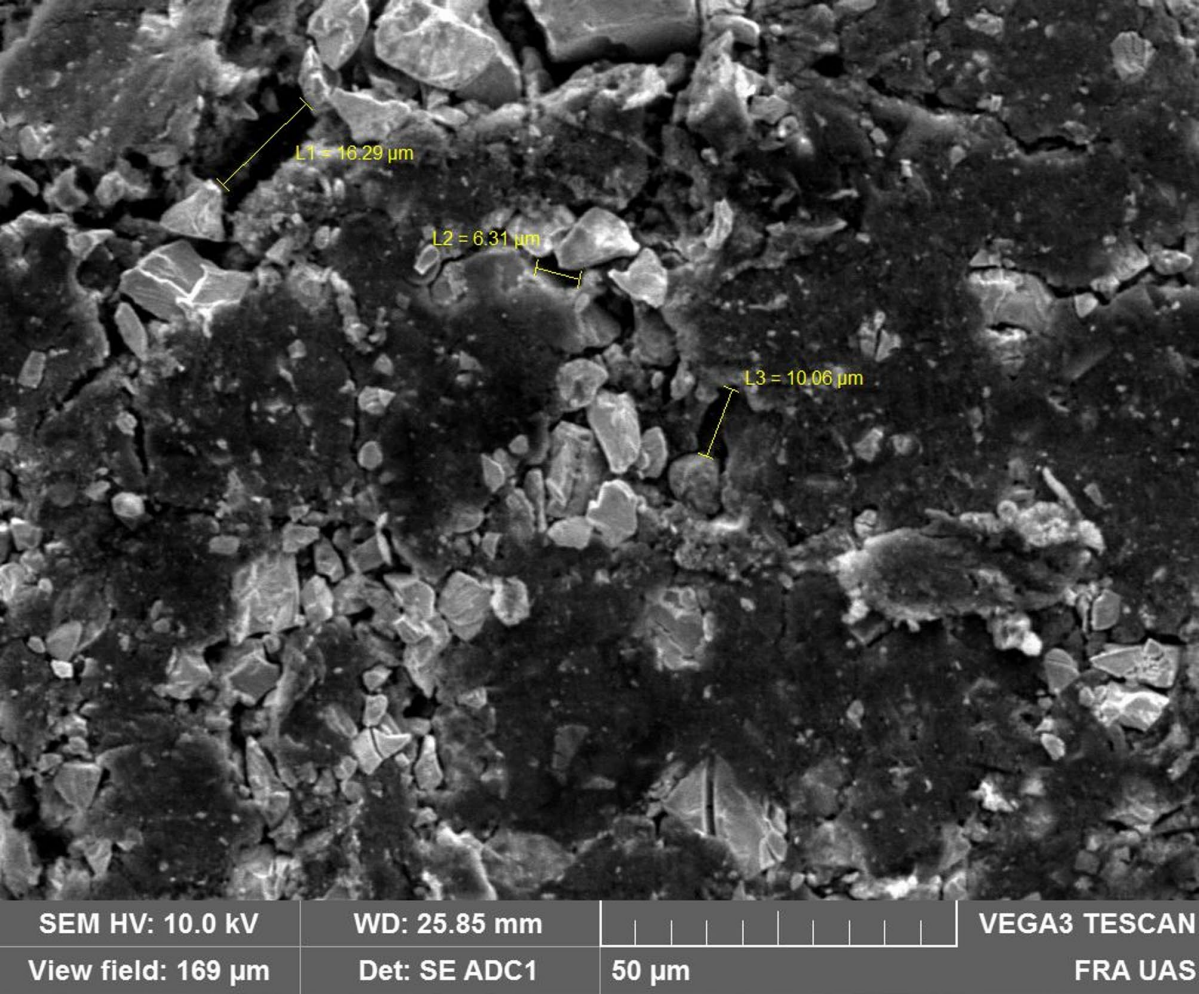
SEM pictures of the electrode structure from NIH33 with 1000x Zoom.

The gas diffusion electrodes created and manufactured by the German company GASKATEL are utilized across all experiments. They are created using a unique mixture of RANEY-Nickel (NI) with polytetrafluoroethylene (PTFE) with a rolling process. The final product is a 0.3 mm thick flexible electrode band. Figure 4 depicts the microscopic electrode’s structural layout. There are shown the Materials, Nickel, PTFE a together and the wholes with a diameter between 5 and 20 μm.

The adjustment of the pore size is decisive for effective mass transfer, since larger micropores result in higher gas flow, particularly for oxygen. The control of water transportation and gas bubble formation become more challenging as the active area shrinks. The use of PTFE prevents the Nickel from flooding and also supports the structure. This type of gas diffusion electrode has been developed to achieve a very high number of three-phase-contact lines, between solid catalyst, liquid electrolyte and gas, which are the regions with very high activity^25,32,33,50,58^. The electrode has a hydrophobic part and a hydrophilic part. To create the optimum hydrodynamic equilibrium, the electrode could be used in different ways by integrating electrodes appropriately under the conditions of the change in Electrolyze the NI. Depending on the PH value and the voltage, the oxidation state from the NI is changing. This has a high influence on the open circuit voltage of the cell and therefore the performance. During operation, the oxidation of the electrodes changes from $\:{Ni}_{3}{O}_{4}$ to a higher state $\:{Ni}_{2}{O}_{3}\:$and raise the potential with them shown in Formula 1. Those is the idle potential in case that there is nothing applied which causes a reduction of the $\:{Ni}_{2}{O}_{3}$.

$$\:2{Ni}_{3}{O}_{4}+{H}_{2}O={3Ni}_{2}{O}_{3}+2H+2{e}^{-}\:\:\to\:\:\:{E}_{0}=1.305-0.0591pH$$

*1:Limit of the domains of relative stability of the substances from Ni in alkane milieu as oxygen reaction according to*^59^.

In case of this oxidation, the voltage increase from every membrane should be the same. If the voltage in idle conditions is below the 1.305 V it indicates that the membrane is not gas stable or that the oxidation of the NIH33 is less.

## Results and discussion

**Fig. 5:**
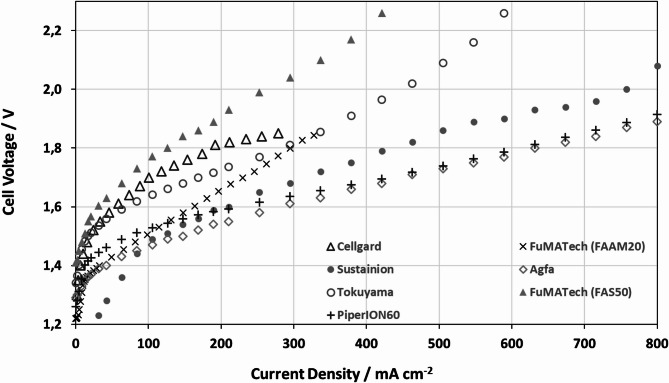
Comparison of different alkaline membranes, separators and AEMs: Agfa UTP220 and Celgard 5550, the alkaline membrane FAAM20 from FuMATech and the AEM- Sustainion X37, Tokuyama A20, PiperION60 and FuMATech FAS 50.

To produce hydrogen under high pressure using non-noble materials as electrodes, we tested various separators, alkaline membranes, and AEMs. Our focus was on the most effective way to produce pure hydrogen with the gas-diffusion electrodes described above. The cell design posed challenges for the separators, especially the no-gap design with the dry hydrogen side, which is atypical for alkane technology. The process efficiency is known to be highly dependent on the operating temperature^50^. Therefore, all membranes were tested at 70 °C to keep uniform operating condition, despite some AEM membranes starting to degrade at this temperature unlike others that were capable of withstanding higher temperatures. Figure 5 compares the performance when two different separators, the UTP220 from AGFA and the Celgard 5550, were used.

The measurements with the separators were conducted with a 6 mol/l-1 KOH solution, but the results between the separators were significantly different. The Agfa UTP220 separator registered the highest efficiency, performed well and is temperature stable. Its power density of up to 800 mA cm^− 2^ at less than 1.9 V is remarkable, but this is only possible because the membrane has satisfactory water transport and a low resistance of only 16 mOhm. However, the Celgard 5550 separator is registering limited power output due to issues with water transport and higher resistance. Both separators, despite the promising power density of the UTP foil, are unable to generate a higher differential pressure as in this case differential pressure as in the range of 2 bar-4 bar. This is due to the general open pore structure of a separator compared to a membrane^41^.

To overcome the problem, the separators were replaced with the alkane membrane FAAM20 from the Fumatech company. As mentioned in the previous paper^50^, the performance of the cell is strongly dependent on the electrode structure, which directly affects the microscopic water and gas management. The membrane’s performance, along with the performance of the two separators, was measured using a 6 mol l^− 1^ KOH solution. The total power output of the cell is rather low in the case of the membrane, at 300 mA cm^− 2^ and 1.85 V. The initial voltage drops below 1.23 V, indicating that the membrane is not gas-stable in this configuration.

So, further testing was carried out with AEM membranes Sustainion X37, Tokuyama A201, Versorgen PiperION60 and the Fumatech FAS 50, which were chosen as they are all suitable for use in a light alkaline medium with sub 1 mol l^− 1^ KOH or even with pure water, so the testing parameters where changed here to 1 mol l^− 1^ KOH. Another test with the Sustainion membrane resulted in a high-power density of up to 750 mA cm^− 2^ with only 2.0 V, but its lifespan and power density are limited by water transport. This AEM shows unstable behaviour during initial operation: thus, it is important to avoid fast power changes or membrane dry out, as this can cause damage, caused by hotspots and a worse conductivity of the membrane. The initial voltage also drops below 1.23 V indicates that the membrane operation is not stable in this configuration at least with higher pressure.

**Fig. 6:**
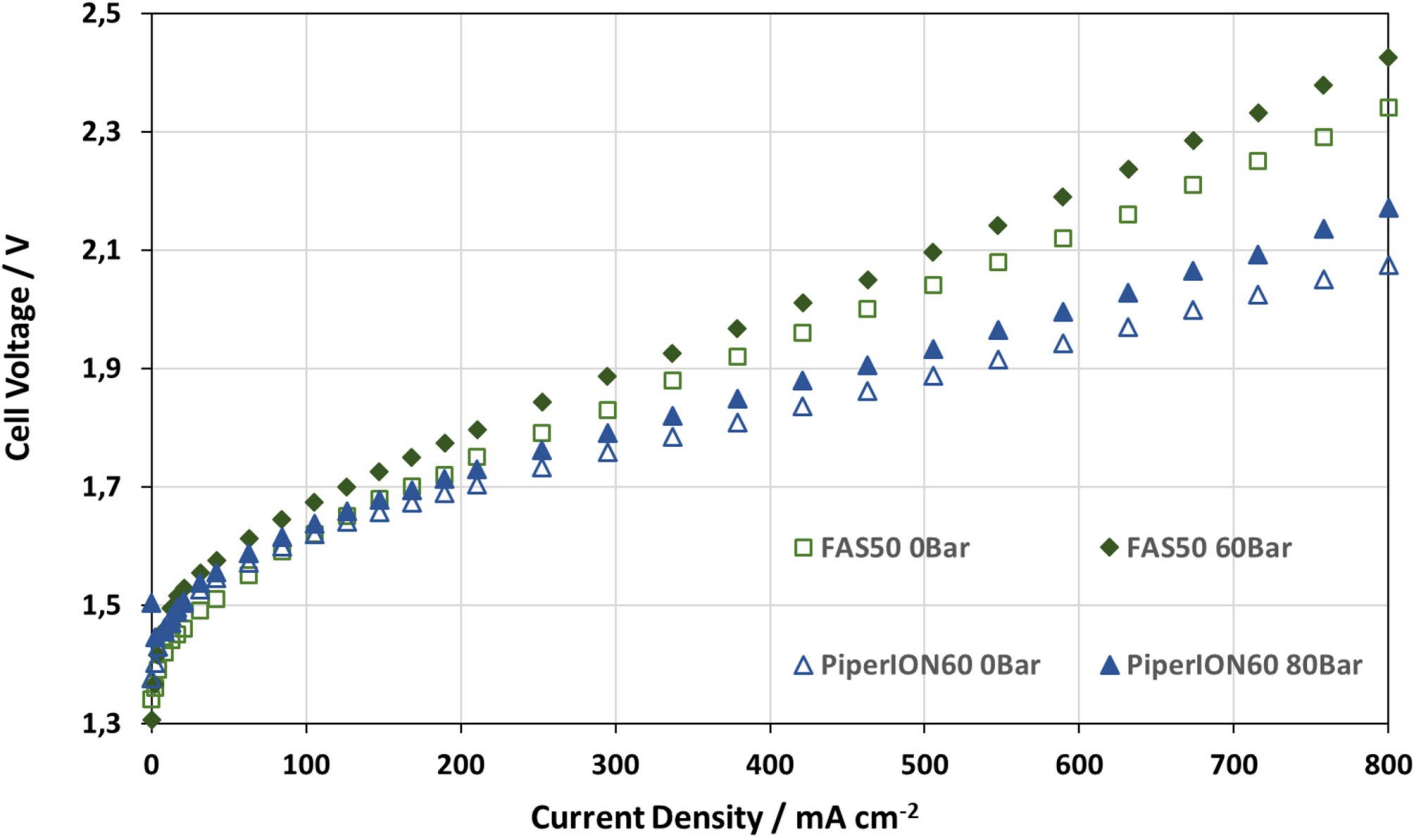
FuMATech **FAS50 and PiperION60** Membrane measured with 1 mol l^-1^ KOH, pretreatment of NaCl with 0 and 60 Bar at 70 °C.

The high gas mixing leads to the formation of an explosive gas, making the operation of this membrane too dangerous for further applications. The A201 from Tokuyama has an output of 350 mA cm^− 2^ at 2.0 V and 2 bars. It is pressure-stable up to 60 bars, even though the power is limited. The open circuit voltage of 1.3 V also indicates a high level of tightness. The FAS 50 and Piperion 60 are the only membranes capable of building up a high differential pressure without any special treatment and with high power densities. It is possible to achieve 500 mA cm^− 2^ with less than 2.0 V. These power density, combined with light KOH and stable, reproducible power, show great promise.

The measured characteristics in Fig. 5 indicate that the FAS 50 and PiperION60 exhibits highly stable pressure behaviour, even at high differential pressures. The idle voltage is above 1.3 V, which is quite high in comparison to the reversible cell of 1.23 V at standard conditions. This is caused by the potential from the Nickel electrode, as described initially. Figure 6 shows the voltage offset between the curves, which is linked to increased pressure. The mean offset value is between 2 bar and 60 bar, with a value of 0.0547 V for the FAS50 and 0.0227 V for the PiperION60.

**Fig. 7:**
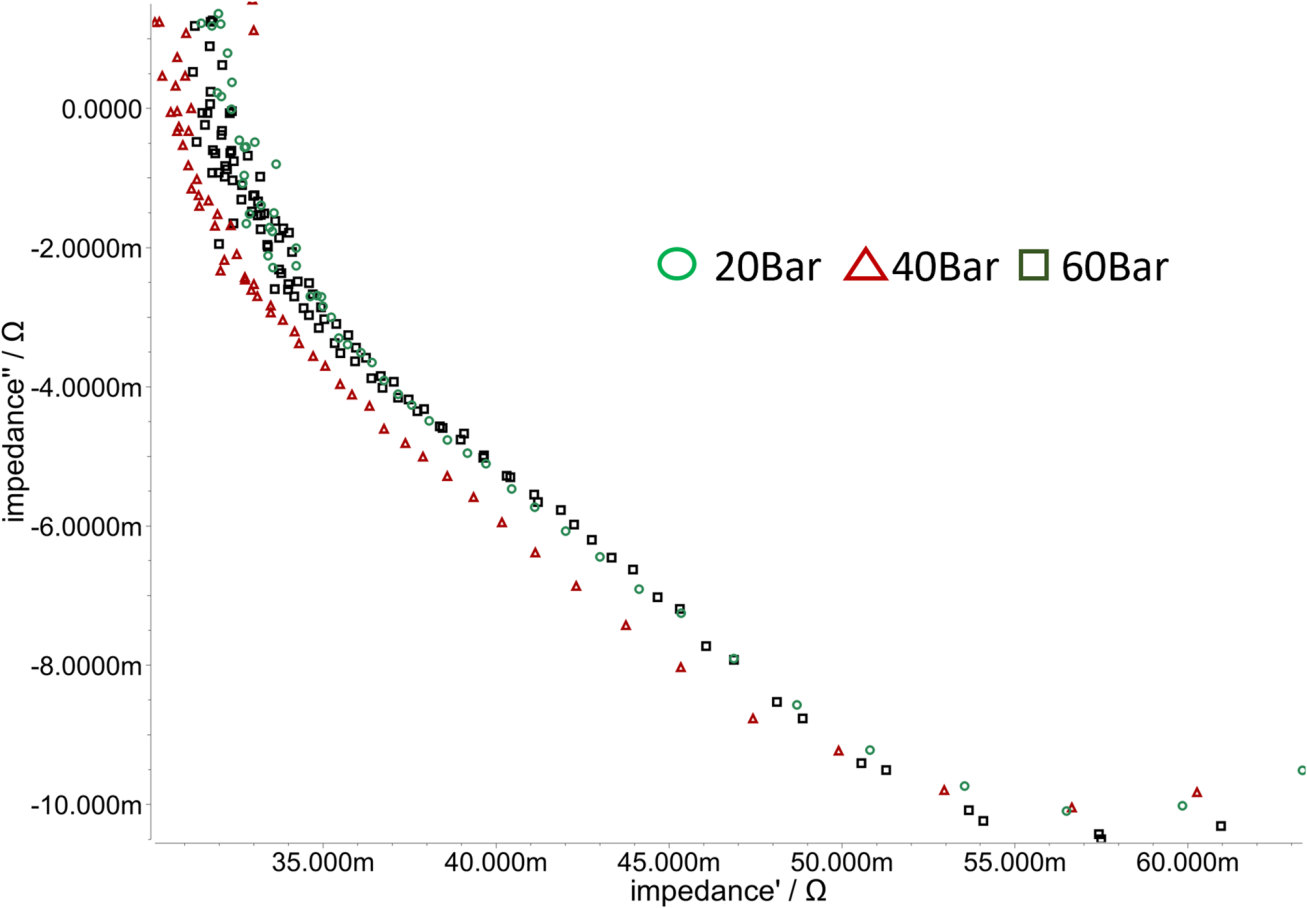
Impedance Spectra in niquist plot from FuMATech **FAS 50** by 20 bar (green circle) 40 bar (red triangle) and 60 bar (black rectangle).

The voltage offset of the FAS50 is also observed in the impedance spectra for the different pressures presented in Fig. 7. The impedance was measured at 125 mA cm^−^², at 70 °C, with 1 mol l^− 1^ KOH. The resistance, measured with the Impedance measured Resistance is 31 mOhm at 20 bar, 30 mOhm at 40 bar, and 31.7 mOhm at 60 bars. For all measurements, the variables that can influence the impedance as in^60^ were kept the same to avoid measurement inaccuracies.

**Fig. 8:**
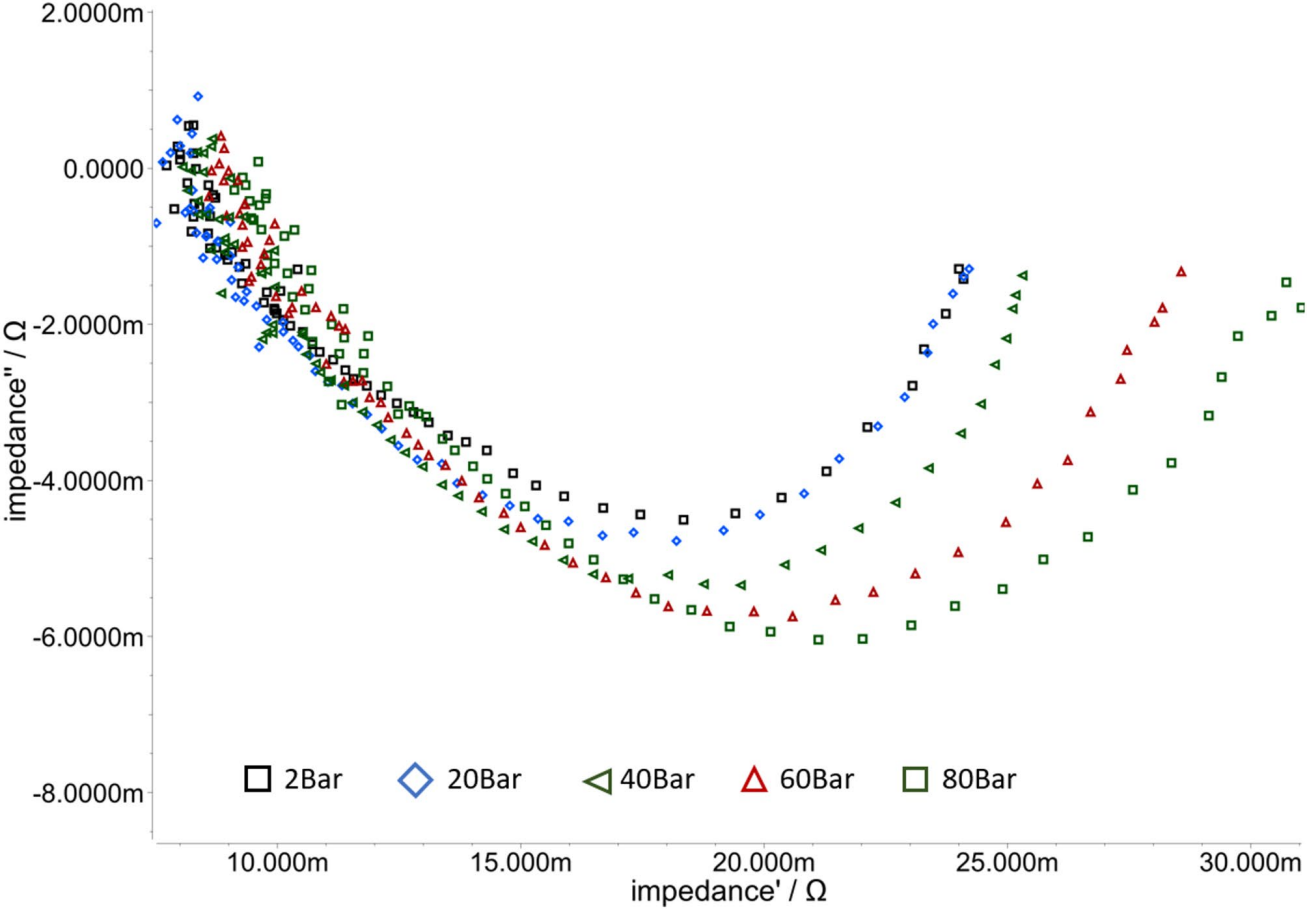
Impedance measurement of the PiperION60 from 2 bar to 80 bar differential pressure with 1 mol/L KOH and 70 °C. 2 bar (black rectangle), 20 bar (Blue rectangle), 40 bar (green triangle), 60 bar (red triangle), 80 bar (green rectangle).

The voltage offset of the PiperION60 is also observed in the impedance spectra for the different pressures presented in Fig. 8. The impedance was measured at 250 mA cm^−^², at 70 °C, with 1 mol l^− 1^ KOH. The resistance, measured with the Impedance measured Resistance is 8.05 mOhm at 0 bar 8,1 mOhm at 20 bar, 8.2 mOhm at 40 bar, 8.6 mOhm at 60 bars and 9.2 mOhm at 80 bar.

In light of the findings that the PiperION exhibited a substantially diminished membrane resistance when confronted with equivalent differential pressure alternatives, it was determined that the utilisation of the PiperION would be pursued with greater fervour within the ambit of the present undertaking. The enhanced efficiency that is concomitantly attained is attributable to the diminished membrane resistance.

**Fig. 9:**
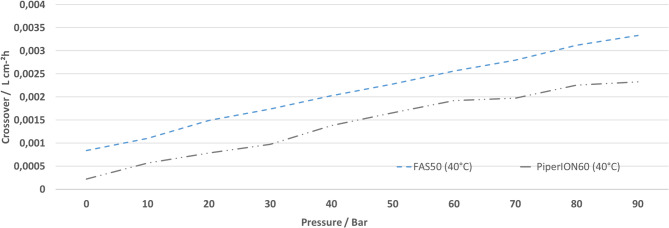
Measurement of the hydrogen crossover of the FAS50 and PiperION60 membranes at 40 degrees as a function of pressure.

In addition to efficiency, which can be readily ascertained from the current voltage characteristic curve, the hydrogen crossover from the HR to the OER side is imperative. This must be considered in both the efficiency analysis and the safety analysis of the entire system. In both analyses, the hydrogen crossover should be minimized. The hydrogen crossover measured in the plant on the OER side is depicted in Fig. 9, standardized to the unit of l cm^− 2^ h^− 1^ to ensure comparability, a necessity that enables the formulation of statements independent of the current.

The characteristic curves demonstrate a clear correlation between hydrogen crossover and pressure, with the PiperION60 exhibiting a lower crossover rate compared to the FAS50. At a current of 500 mA cm^−^², equivalent to approximately 1.9 V, the PiperION60 maintains below 50% of the LEL up to 80 bar. In contrast, the FAS 50 surpasses this threshold, achieving 75% LEL and a voltage of 2.08 V at the same current. These findings reiterate the hypothesis that the PiperION60 is particularly well-suited for high-pressure electrolysis, as it exhibits enhanced efficiency and reduced safety concerns due to its diminished crossover.

With the measured data it is possible to simulate the behaviour inside the electrolyser cell. Depending on the frequency of the impedance the behaviour of the membrane, the electrodes and the mass transport could be estimated. The model is used to describe the electrochemical process including both electrodes and the membrane and is shown in Fig. 2.

**Fig. 10:**
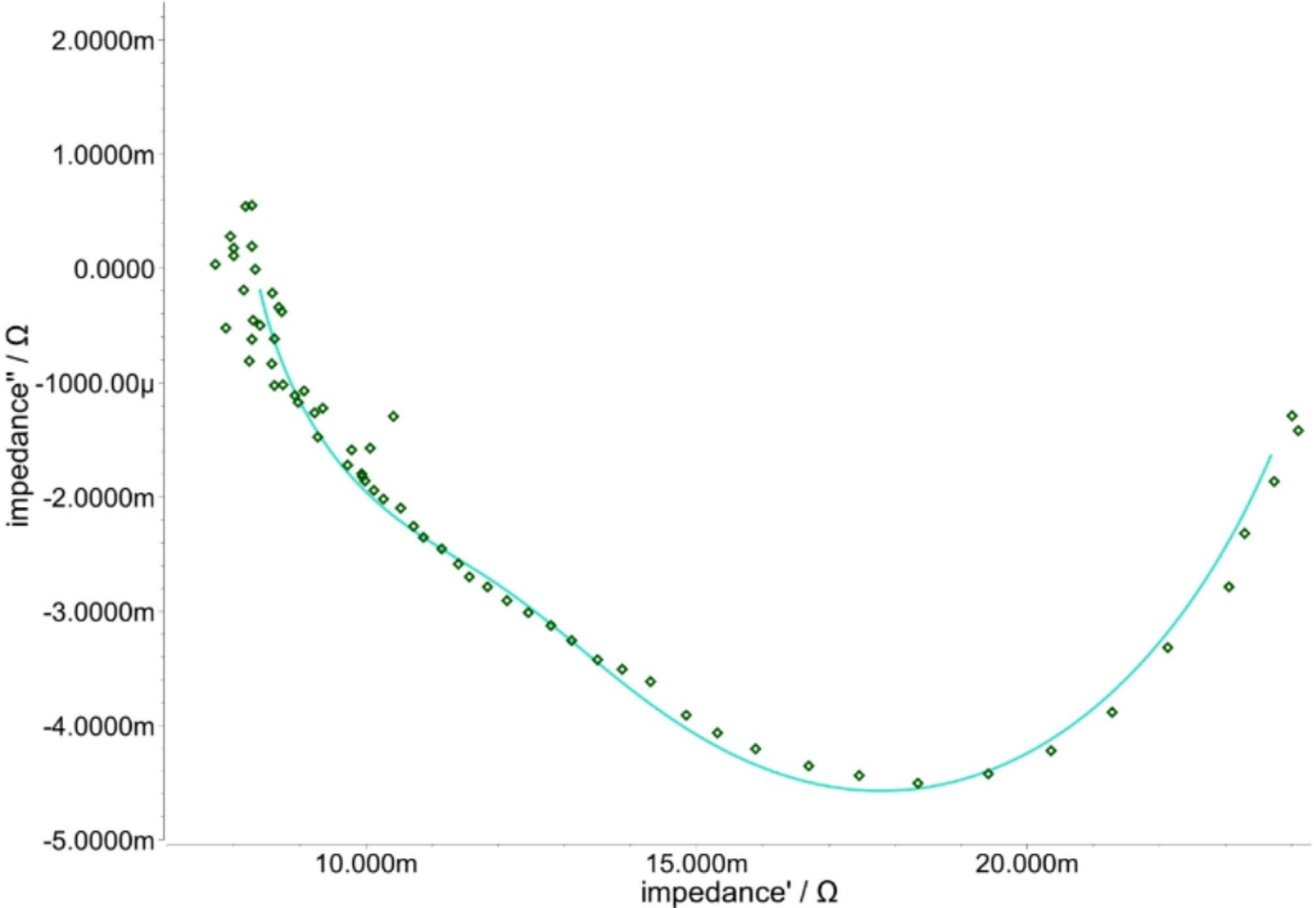
Impedance plot for Piperion60 at 00 bar. The rectangle points represent the measured values, while the lines represent the simulated values.

The trend was generated from the model outputs as shown in Fig. 10. It shows the comparison between the simulation data with the measured data. The rectangle points represent measured impedance and the curve represents simulation trend. The simulation was carried out for all the measured impedances ranging from 0 bar to 80 bar. It is important to note that the 0- bar measurement was used solely as an example. In the model a simple ohmic resistance for the membrane and complex resistances for each electrode are used, as shown in Fig. 2. To explain the complex multi-physics behaviour of the porous Nickel structure, a constant phase element is required for the electrode. The constant phase element (CPE) is an important electrochemical modelling element used in the characterization of electrode systems^51–53^. In contrast to an ideal capacitor, the CPE represents a non-ideal, frequency-dependent capacitance and enables a more precise description of diffusion processes and non-linearity`s on electrode surfaces. Its complex impedance behaviour is represented by a phase angle (α) that can better represent the dynamic electrode processes. The value α = 1 gives an ordinary capacitor, α = 0 is a resistor, and α = 0.5 corresponds to the Warburg element used to model diffusion processes. Table 3 presents the results of the simulation model for impedance measurements conducted at 0, 20, 40, 60, and 80 bars.

**Table 3:** Simulation results of the EIS measurement for the PiperION60 from 00 to 80 bar.

Pressure	HER electrode	Membrane	OER electrode	Error of Simulation
00 bar	V = 750 mF α = 813 m *R* = 3.56 mOhm	*R* = 8.05 mOhm	V = 2200 mF α = 763 m *R* = 12.7 mOhm	3.77%
20 bar	V = 1600 mF α = 983 m *R* = 1.8 mOhm	*R* = 8.10 mOhm	V = 763 mF α = 701 m *R* = 15.3 mOhm	4.4%
40 bar	V = 2630 mF α = 1120 m *R* = 1.09 mOhm	*R* = 8.22 mOhm	V = 496 mF α = 682 m *R* = 17.4 mOhm	3.27%
60 bar	V = 522 mF α = 986 m *R* = 3.13 mOhm	*R* = 8.65 mOhm	V = 407 mF α = 642 m *R* = 18.2 mOhm	3.24%
80 bar	V = 622 mF α = 930 m *R* = 1.86 mOhm	*R* = 9.2 mOhm	V = 375 mF α = 668 m *R* = 20.5 mOhm	2.29%

The results are divided into columns for the three main simulation groups: membrane, HER and OER. The specified error indicates the deviation of the simulated values from the measured points. The values of the hydrogen electrode exhibit only slight changes with increasing pressure. The membrane resistance fluctuates slightly with pressure changes, from 8.05 mOhm to 9.2 mOhm. The capacitor values V of the HER varies with increasing pressure and someuncertainty. The phase angle α fluctuates from 813 m to 1120 m, resistance fluctuates from 3.56 mOhm to 1.8 mOhm, and V fluctuates from 522 mF to 2.62 F. The maximum failure in the simulation is 4.4% for the 20- bar characteristic curve.

The increase in membrane resitance R from 8.05 mOhm to 9.2 mOhm resistance should be linked to water transport limitations and could also be caused by membrane pressure in addition to contact changes caused by the pressure.

Water transport through the cell is a critical component. The rise in resistance R and decrease in capacity V indicate a change in the behaviour on the OER. The resistance is rising from 12.7 mOhm up to 20.5 mOhm. Thereby, the decrease of the phase angle α from 763 to 668 suggests a change in transport behaviour from a capacitor more to a Wartburg Element. It means that the transport and oxygen evolution issues by higher pressure rising.

This change towards a Warburg element can be explained with distinctive diffusion problems at the oxygen electrode, where gas bubbles are formed in liquid electrolyte. A higher pressure difference across the membrane could lead to strong back diffusion of hydrogen gas which disturbs especially the mass transport at the OER.

**Fig. 11:**
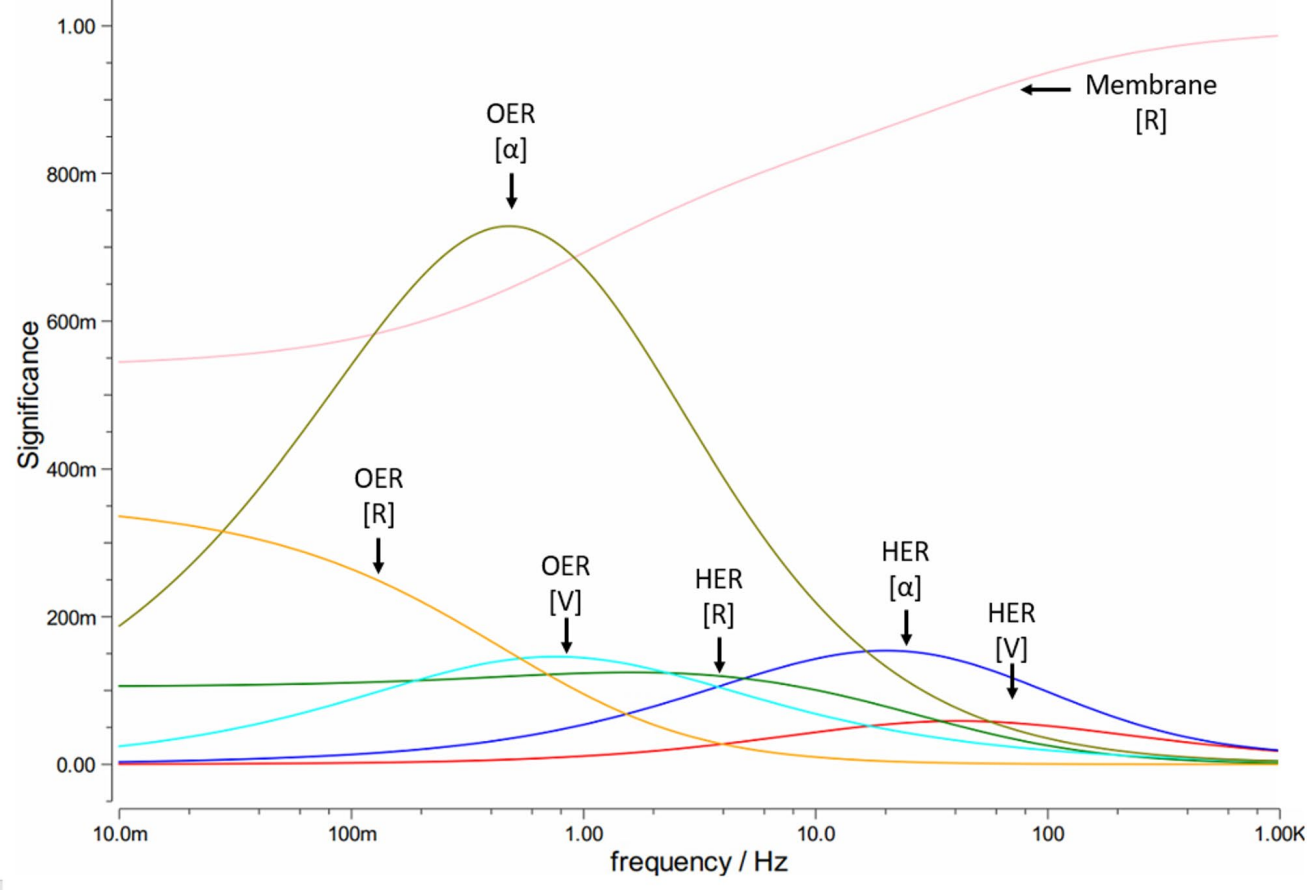
Significance plot from simulation of FAS30 20 bar impedance measurement.

Additionally, in Fig. 11 the significants of the simulation as a function of the excitation frequency are shown. As given in literature^61^, it can be seen that the dynamic operational conditions are at play^61^. This significance describes the influence of the individual simulation constants on the resulting graph at various frequencies of the impedance measurement. The hydrogen electrode is described by HER [α; V; R], which have a smaller influence on the curve and which are mostly focused on high frequencies. The OER [α; V; R] describe the oxygen electrode and the mass transport, respectively. The simulation correlates reasonable to the measured data. Thus, we could show that that the membrane and OER have the greatest influence on the electrolyzer’s performance. Especially the high significance of the OER phase angle (see Fig. 11) is remarkable. The peak is located around 0.7 Hz which is in the range of the bubble detachment frequency. This could be taken as an evidence for the high complexity of the bubble formation process in liquid solution, represented by the characteristic of the Wartburg element. In general, Fig. 11 shows that increasing pressure difference negatively affects the behaviour of the mass transport system at the OER and membrane, reducing performance. In the near future, numerical simulations of the porous electrode structure will give more insides into the complex physical phenomena especially at the OER.

However, it is important to note that the achieved pressures of 80 bars at this power density are noteworthy. As a differential pressure with a ‘dry’ HER electrode, these research results are unique and offer great potential for future applications.

## Conclusion

One pathway to reduce the overall costs of renewable hydrogen and increase the reliability of electrolyzer plants is to increase the operating pressure of water electrolyzers, which limits the need for external mechanical compression. Another significant cost driver are the manufacturing costs of the electrolysis plant. Anion exchange electrolysis is a less expensive alternative to PEM technology for producing pre-compressed hydrogen. Concerning to the PFAS problematic of PEM, the AEM also have a greater potential in terms of environmental compatibility.

This work presents a potential design of a high-pressure electrolyzer with anion exchange membrane (AEM)was experimentally investigated - with very promising results. To produce dry hydrogen under high differential pressure, several different alkaline membranes and anion exchange membranes were investigated. The use of a non-gap design and the AEM PiperION60 allow to produce pure hydrogen at pressures up to 80 bars, with a current density of 500 mA cm^− 2^ at 2.0 volts. The high hydrogen pressure, requires only a slight voltage offset, resulting in a in high efficiency and therewith improved total efficiency of hydrogen storage systems. The simulation Model clearly shows the way for future improvements on this technology which will have a huge impact on performance end efficiency. Future work aims to achieve greater power output while using the same area and lower cell voltage. The simulation makes it possible to gain further insights into the processes that take place in the electrolyser and to identify and understand the optimization potential. Especially the oxygen evolution with bubbles in flooded electrodes seem to be the crucial point. This will be achieved through the optimization of electrodes, resulting in a larger active area and lower resistance.

## Data Availability

The datasets used and/or analysed during the current study available from the corresponding author on reasonable request.
